# Emergence and characteristics of multidrug-resistant *Salmonella enterica* subspecies *enterica* serovar Infantis harboring the pESI plasmid in chicken slaughterhouses in South Korea

**DOI:** 10.1128/spectrum.02955-24

**Published:** 2025-06-02

**Authors:** Jiyeon Jeong, Myeongju Chae, Min-Su Kang, Ji-Youn Lee, Yong-Kuk Kwon, Hye-Jin Lee, So-Hee Lee, Hyeon-Bin Son, Jin-San Moon, Seongbeom Cho

**Affiliations:** 1 Avian Disease Research Division, Animal and Plant Quarantine Agency 65359, Gimcheon, Gyeongsangbuk, South Korea; 2 College of Veterinary Medicine and Research Institute for Veterinary Science, Seoul National University 26725, Seoul, South Korea; University of Maryland Eastern Shore, Princess Anne, Maryland, USA

**Keywords:** *Salmonella *Infantis, pESI, ST32, multidrug resistance, *bla_CTX-M-65_*, chicken slaughterhouses

## Abstract

*Salmonella enterica* subsp. *enterica* serovar Infantis (*S*. Infantis), a major cause of human salmonellosis, is commonly associated with transmission via contaminated chicken meat. This study, as part of the national *Salmonella* monitoring program, assessed the prevalence of *S. enterica,* including *S*. Infantis, in chicken slaughterhouses across South Korea from 2014–2022. The presence of a megaplasmid, known as plasmid of emerging *S*. Infantis (pESI), was confirmed. This confirmation was based on multidrug-resistant and third-generation cephalosporin-resistant *S*. Infantis isolates using whole-genome sequencing. Phenotypic and genotypic characterization involved antimicrobial susceptibility tests, pulsed-field gel electrophoresis, polymerase chain reaction to screen for pESI plasmids, plasmid profiling, and conjugation assays. *S*. Infantis was identified in 9.3% of *Salmonella*-positive samples in 2014, undetected from 2015–2020, but re-emerged as the predominant serovar in 2021 (54.7%) and 2022 (75.5%). The isolates in 2014 were antibiotic susceptible, whereas most isolates from 2021 to 2022 exhibited multidrug-resistance, including resistance to third-generation cephalosporins. All isolates were sequence type 32 (ST32), with core genome multilocus sequence typing demonstrating pESI plasmid-based clustering. The pESI^+^ isolates harbored genes, such as *aadA1*, *dfrA14*, *sul1*, and *tetA*, and three multidrug-resistant pESI^+^ isolates harbored *bla_CTX-M-65_*. The plasmids were genetically similar to those observed in *S*. Infantis from broilers, chicken meat, and human clinical samples across various countries. This study highlights the spread of multidrug-resistant *S*. Infantis harboring the pESI plasmid with *bla_CTX-M-65_* during early chicken production in South Korea. Continuous monitoring and control of resistant *S*. Infantis throughout the food chain is essential to inform public health initiatives.

**IMPORTANCE:**

This study highlights the critical emergence of multidrug-resistant (MDR) *Salmonella enterica* serovar Infantis (*S*. Infantis) in South Korea’s chicken slaughterhouses, driven by the acquisition of the pESI megaplasmid harboring the extended-spectrum beta-lactamase (ESBL) determinant *bla_CTX-M-65_*. Using whole-genome sequencing and comprehensive phenotypic-genotypic analyses, the findings reveal that pESI^+^ isolates in South Korea are genetically similar to strains from broilers, chicken meat, and human clinical cases worldwide. This underscores the transboundary nature of *S*. Infantis and its potential as a global public health threat.

## INTRODUCTION

Salmonellosis is a prevalent food-borne disease and complex zoonosis that affects global public health. Over 2,600 serotypes of *Salmonella* are known; however, a limited number are associated with numerous human diseases, and the prevalence of different serovars may fluctuate over time (1). Among these, *Salmonella enterica* subsp. *enterica* serovar Infantis (*S*. Infantis) is a poultry-adapted serovar that has been increasingly reported in broiler chickens and frequently identified in human salmonellosis cases worldwide (2–6). Broiler chickens serve as primary reservoirs of *S*. Infantis, contributing to meat contamination during slaughter in slaughterhouses (1). Therefore, the consumption of chicken meat is considered a risk factor for human infection with *S*. Infantis.

In recent years, in addition to its increasing prevalence, *S*. Infantis from various animal sources and humans worldwide has exhibited antimicrobial resistance (3, 7–9). The increasing incidence and dissemination of various multidrug-resistant (MDR) *S*. Infantis clones in broiler populations have resulted in their transmission to humans through the food chain and poultry products in countries such as Hungary (10), Italy (11), Switzerland (12), Slovenia (13), and Russia (14).

The emergence of extended-spectrum beta-lactamase (ESBL)-producing strains has increased worldwide (15–17). Third-generation cephalosporins have been used to treat severe salmonellosis. Therefore, the strains exhibiting resistance to these drugs have limited treatment options (18). The beta-lactamase cefotaximase type M (*bla_CTX-M_*) gene is the most widely distributed ESBL gene that confers resistance to third-generation and other cephalosporins. *S*. Infantis strains containing *bla_CTX-M-65_* have been detected worldwide (11, 12, 19, 20). A significant factor for the increased prevalence, MDR, and ESBL production of this serovar has been the acquisition of the plasmid of emerging *S*. Infantis (pESI)-like conjugative megaplasmid, initially described in an Israeli strain of *S*. Infantis (21) and later identified globally (11, 22, 23). This plasmid (approximately 300 kbp in size) harbors numerous antimicrobial resistance genes (ARGs), including the *bla_CTX-M_* genes responsible for the production of ESBL enzymes. Additionally, it has numerous virulence factors that confer a survival advantage over other *Salmonella* strains, such as the yersiniabactin iron acquisition operon. Moreover, the plasmid contains several fimbriae that facilitate increased attachment to human and poultry epithelial cells (21, 24).

Whole-genome sequencing (WGS) is essential for tracking the antibiotic resistance-related genetic characteristics of this plasmid in *S*. Infantis, and the spread and genetic alterations of the pESI plasmids in the food chain and among various hosts. To gain a detailed understanding of pESI plasmids, it is essential to complement short-read next-generation sequencing with long-read sequencing technologies, owing to the large size of these plasmids. However, few studies have reconstructed the complete gap-free sequences of pESI using these hybrid approaches (7, 17, 25, 26).

Owing to the global trend of increasing MDR *S*. Infantis, we assessed the prevalence, antibiotic resistance profiles, and genetic characteristics of *S*. Infantis in chicken slaughterhouses across South Korea. Additionally, we determined the emergence of pESI-harboring *S*. Infantis using WGS to analyze the genetic features of *S*. Infantis isolates, focusing on their epidemiology and antibiotic resistance. Moreover, we used an advanced hybrid assembly to obtain complete sequences of the representative pESI plasmids and compared them with publicly available pESI sequences from other countries, aiming to understand their global distribution and effects.

## MATERIALS AND METHODS

### Sample collection

As part of a South Korean national study for monitoring *Salmonella* in chicken slaughterhouses conducted by the Animal and Plant Quarantine Agency, samples were collected from 51 slaughterhouses across 10 South Korean provinces (Gyeonggi, Gangwon, Chungbuk, Chungnam, Gyeongbuk, Gyeongnam, Jeonbuk, Jeonnam, Daegu, and Jeju) between May 2014 and December 2022. A total of 1,554 samples were collected along the slaughter line, with one flock selected from each slaughterhouse. These samples included cloacal swabs from live birds (*n* = 30), feces from crates (*n* = 82), shackles (*n* = 42), as well as floor and wall surfaces in the bleeding area (*n* = 36), carcasses before/after scalding (*n* = 36), scalding water (*n* = 14), carcasses after defeathering (*n* = 30), feathers (*n* = 56), carcasses before/after evisceration (*n* = 306), worker gloves (*n* = 30), workstation surfaces of the evisceration step (*n* = 30), chilling water (*n* = 59), floor and wall surfaces in the air-chilling room (*n* = 6), workstation and wall surfaces in the grading and packaging room (*n* = 38), and carcasses after chilling (final carcasses) (*n* = 759). All the samples were transported to the laboratory in an icebox within 24 h and tested for *Salmonella* contamination immediately after their arrival.

### *Salmonella* isolation and serotyping

Sample preparation was performed, as previously described (27). Swab suspension (5 mL) and carcass rinsate (30 mL) were inoculated into corresponding volumes of buffered peptone water (BD Biosciences, Sparks, MD, USA). Subsequently, they were incubated at 37℃ for 24 h and transferred into tetrathionate broth (BD Biosciences) and Rappaport-Vassiliadis broth (Merck, Darmstadt, Germany). The cultures were incubated at 37℃ for 24 h and streaked onto xylose lysine deoxycholate agar (BD Biosciences) and Rambach agar (Merck) plates, followed by further incubation at 37℃ for 24 h (28). *Salmonella* was identified based on colony morphology and amplification of the invasion A (*invA*) gene by polymerase chain reaction (PCR) (29). The serotypes of the isolates were determined using O and H antisera (BD Diagnostics), following the Kauffmann-White scheme (30). The annual prevalence of *S. enterica* and serovar Infantis was compared using the Chi-square test with SigmaPlot 14.0 (Systat Software Inc., San Jose, CA, USA), and differences were considered statistically significant at *P* < 0.05.

### Antimicrobial susceptibility testing

Antimicrobial susceptibility testing was performed on all 191 *S*. Infantis isolates. Susceptibility to 14 antibiotics from eight classes was assessed by determining the minimum inhibitory concentrations (MICs) using the broth microdilution method with Sensititre (Trek Diagnostic Systems, Cleveland, OH, USA) in accordance with the manufacturer’s protocol. The antibiotics tested, which are crucial for public health and commonly used in treating *Salmonella* infections in poultry, included amoxicillin-clavulanic acid (1/0.5 to 32/16 mg/L), ampicillin (1–32 mg/L), azithromycin (0.12–8 mg/L), cefoxitin (0.5–32 mg/L), ceftiofur (0.12–8 mg/L), ceftriaxone (0.25–64 mg/L), chloramphenicol (2–16 mg/L), ciprofloxacin (0.015–4 mg/L), gentamicin (0.25–16 mg/L), nalidixic acid (0.5–16 mg/L), streptomycin (2–64 mg/L), sulfisoxazole (16–256 mg/L), tetracycline (4–32 mg/L), and trimethoprim-sulfamethoxazole (0.12/2.38 to 4/76 mg/L). MICs were interpreted following the guidelines of the Clinical and Laboratory Standards Institute guidelines (31) or the National Antimicrobial Resistance Monitoring System (32).

### Pulse-field gel electrophoresis

Pulse-field gel electrophoresis (PFGE) was performed on 95 out of 191 *S*. Infantis isolates, excluding duplicate strains with identical antimicrobial resistance patterns from the same source, slaughterhouse, and sampling period, using restriction endonuclease XbaI (33). Gel images were analyzed using the BioNumerics software (Applied Maths, Sint-Martens-Latem, Belgium). A dendrogram was generated from the Dice coefficients of similarity using the unweighted-pair group method with average linkages (UPGMA).

### Identification of pESI plasmid and conjugation assay

To confirm the presence of the pESI plasmid, 38 strains, each representing a PFGE type, were selected for PCR screening and plasmid profiling. To detect the pESI plasmid in the isolates, a PCR screening for three targets encoding the plasmid backbone (hyp pESI) and fimbriae (Fim and K88) was conducted (21). The location of the pESI plasmid was determined using plasmid profiling (34).

pESI plasmid was transferred by conjugation using the liquid mating method (35) using the 30 *S*. Infantis isolates resistant to one or more antibiotics as donors, and rifampin-resistant *Escherichia coli* RG488 was used as the recipient strain. Considering that 29 out of 30 isolates were resistant to ceftiofur and 1 isolate was resistant to nalidixic acid, we selected the transconjugants on MacConkey agar (BD Biosciences) plates supplemented with ceftiofur or nalidixic acid (for pESI selection) and rifampin (for recipient strain selection). To confirm the presence of the megaplasmid in the transconjugants, colony PCR was conducted to amplify three targets (hyp pESI, Fim, and K88).

### WGS and data analysis

WGS was performed on seven representative isolates selected based on PFGE pattern, phenotypic and genotypic characteristics, isolation source, and isolation year, using the Illumina MiSeq platform (Illumina Inc., CA, USA) and Oxford Nanopore sequencing platform (Oxford Nanopore Technologies Limited, Oxford, UK). Library preparation and DNA sequencing were performed by a commercially available company (Sanigen, Anyang, Republic of Korea). Briefly, Illumina and Nanopore sequencing data were processed for quality control and subjected to *de novo* assembly using Unicycler v0.4.8 (36), implemented in the Pathosystems Resource Integration Center (PATRIC) v3.6.12 Web Server. Subsequently, the genome was annotated using RASTtk (37). The assembled sequences were analyzed to confirm the species and *Salmonella* serotype using Center for Genomic Epidemiology (CGE) pipelines: KmerFinder (version 2) (38) and SeqSero (version 1.1). After confirmation, the multilocus sequence typing (MLST) results for *Salmonella enterica*, plasmid incompatibility group, acquired ARGs, and virulence-associated genes were identified using the pipelines: MLST (v1.7) (39), PlasmidFinder (v1.2) (40), ResFinder (v2.1) (41), and Virulence Factor Database (v5.0) (42), respectively, with 98% identity and 60% minimum alignment length as thresholds, that are also available from the CGE. Additionally, pMLST of the pESI plasmid was performed using pMLST (v2.0) (40) according to the Incl1 pMLST scheme. We conducted a comparative genomic analysis using the core-genome MLST (cgMLST) between our isolates and 94 additional *S*. Infantis genomes registered in the public databases (NCBI or ENA; data collected on 20 December 2023) listed in Table S1. These 94 genomes encompass the genomes of isolates obtained from broilers, chicken meat, and human sources from 2006 to 2021 in 14 countries, including a representative pre-emergent *S*. Infantis isolate (Strain Id 335-3, Israel, 1970, Human) and an initially reported emergent isolate (Strain ID119944; Israel, 2008, Human), as registered in published papers on emerging *S*. Infantis (ESI) (11, 12, 14, 17, 21, 22, 26, 43–45). For cgMLST analysis, we used the *Salmonella* scheme from EnteroBase comprising 3002 loci (46) (https://enterobase.warwick.ac.uk/). The circular cgMLST tree was created using the neighbor-joining algorithm in Grapetree (47), and subsequently annotated and visualized using iTOL v6.3 (48). The cgMLST minimum spanning tree was constructed using the Grapetree v1.5.0 (47) and MSTreeV2 algorithm. The four reconstructed Korean pESI plasmids were compared with the public sequences listed in Table S2. Pangenome analysis of pESI sequences was performed using GView Server v1.7 (36). Average nucleotide identity using MUMmer (ANIm) values were calculated using JSpeciesWS (70).

## RESULTS

### Increasing prevalence and antimicrobial resistance rates of *S*. Infantis isolates

**Fig 1:**
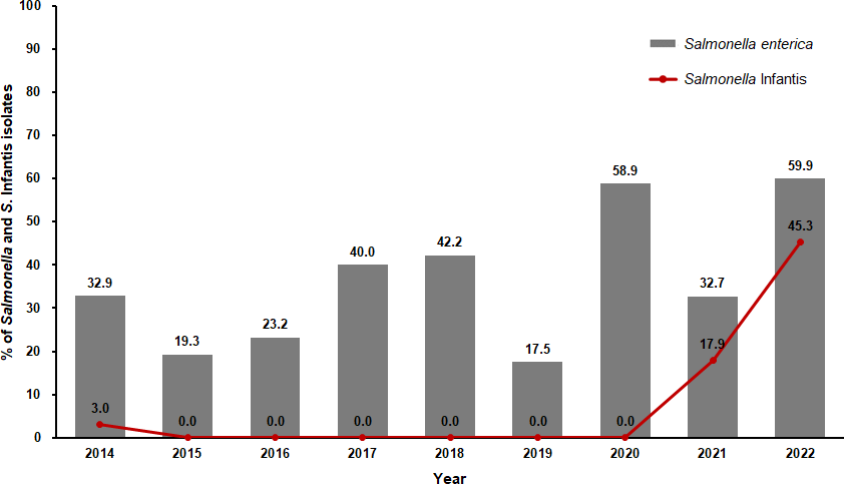
Prevalence of *Salmonella enterica* and serovar Infantis in chicken slaughterhouses in South Korea during 2014–2022.

From May 2014 to December 2022, 581 out of 1,554 samples (37.4%) from 51 chicken slaughterhouses were positive for *Salmonella*. Among these, 191 (32.9%) were identified as *S*. Infantis (Table S3). *Salmonella* isolation rates varied (17.5%–59.9%), with the prevalence of *S*. Infantis among tested samples and *Salmonella*-positive samples being 3.0% and 9.3% in 2014, not detected from 2015 to 2020, and increased to 17.9% and 54.7% in 2021, and 45.3% and 75.5% in 2022, respectively (Fig. 1; Table S3).

In the 191 *S*. Infantis isolates, the highest resistance percentage was observed with ampicillin (89.5%), followed by nalidixic acid (89.0%), ceftiofur (88.5%), ceftriaxone (88.5%), sulfisoxazole (88.5%), and tetracycline (88.5%). Twenty patterns of antibiotic resistance were recognized (Table 1). In 2014, all the five isolates were susceptible to all the tested antibiotics, whereas in 2021 and 2022, 36 (76.6%) and 113 (81.3%) isolates, respectively, were resistant to seven or more classes of the antimicrobials tested (Table 1). The MDR rates of *S*. Infantis isolates were 0%, 87.2%, and 93.5% in 2014, 2021, and 2022, respectively, with significant differences between 2014 and 2021/2022 (*P* < 0.05) (Table 1).

**TABLE 1:** Antimicrobial resistance patterns of 191 *S*. Infantis isolated from chicken slaughterhouses^*a*^^,^^*c*^

Resistance pattern	No. of antimicrobial classes	No. (%) of isolates		
		2014 (*n* = 5)	2021 (*n* = 47)	2022 (*n* = 139)
AUG-AMP-AZI-**XNL-AXO**-CHL-NAL-STR-FIS-TET-SXT	8	0 (0)	0 (0)	1 (0.7)
AMP-AZI-**XNL-AXO**-CHL-GEN-NAL-STR-FIS-TET-SXT	8	0 (0)	0 (0)	2 (1.4)
AMP-AZI-**XNL-AXO**-CHL-NAL-STR-FIS-TET-SXT	8	0 (0)	0 (0)	8 (5.8)
AMP-**XNL-AXO**-CHL-CIP-NAL-STR-FIS-TET-SXT	8	0 (0)	0 (0)	1 (0.7)
AMP-**XNL-AXO**-CHL-GEN-NAL-STR-FIS-TET-SXT	7	0 (0)	9 (19.1)	28 (20.1)
AMP-FOX-**XNL-AXO**-CHL-NAL-STR-FIS-TET-SXT	7	0 (0)	1 (2.1)	3 (2.2)
AMP-AZI-**XNL-AXO**-CHL-NAL-FIS-TET-SXT	7	0 (0)	0	1 (0.7)
AMP-**XNL-AXO**-CHL-GEN-NAL-STR-FIS-TET	7	0 (0)	2 (4.3)	2 (1.4)
AMP-**XNL-AXO**-CHL-NAL-STR-FIS-TET-SXT	7	0 (0)	**23** (**48.9**)	**64** (**33.1**)
AMP-FOX-**XNL-AXO**-CHL-NAL-STR-FIS-TET	7	0 (0)	0 (0)	1 (0.7)
AMP-**XNL-AXO**-CHL-NAL-STR-FIS-TET	7	0 (0)	2.1	2 (1.4)
AMP-**XNL-AXO**-GEN-NAL-STR-FIS-TET-SXT	6	0 (0)	4.3	0 (0)
AMP-**XNL-AXO**-GEN-NAL-STR-FIS-TET	6	0 (0)	0 (0)	14 (10.1)
AMP-**XNL-AXO**-CHL-NAL-FIS-TET-SXT	6	0 (0)	0 (0)	1 (0.7)
AMP-**XNL-AXO**-NAL-STR-FIS-TET-SXT	6	0 (0)	1 (2.1)	1 (0.7)
AMP-CHL-NAL-FIS-TET-SXT	5	0 (0)	1 (2.1)	0 (0)
AMP-**XNL-AXO**-CHL-NAL	4	0 (0)	0 (0)	1 (0.7)
STR-TET-SXT	3	0 (0)	1 (2.1)	0 (0)
NAL	1	0 (0)	1 (2.1)	0 (0)
Pan-susceptible^*b*^	0	**5 (100**)	5 (10.6)	9 (6.5)
**MDR (≥3 antimicrobial classes)^*b*^**		**0** (**0**)	**41** (**87.2**)	**130** (**93.5**)

^*a*^ AMP, ampicillin; AUG, amoxicillin-clavulanic acid; AXO, ceftriaxone; AZI, azithromycin; CHL, chloramphenicol; CIP, ciprofloxacin; FIS, sulfisoxazole; FOX, cefoxitin; GEN, gentamicin; NAL, nalidixic acid; STR, streptomycin; SXT, trimethoprim-sulfamethoxazole; TET, tetracycline; XNL, ceftiofur; MDR, multi-drug resistance.

^*b*^ There was a significant difference (*P* < 0.05) in the number of *S*. Infantis exhibiting the corresponding antibiotic resistance pattern in 2014, 2021, and 2022.

^*c*^ The highest proportion is indicated in bold.

### Comparative genetic distribution of *S*. Infantis isolates and acquisition of pESI plasmids

**Fig 2:**
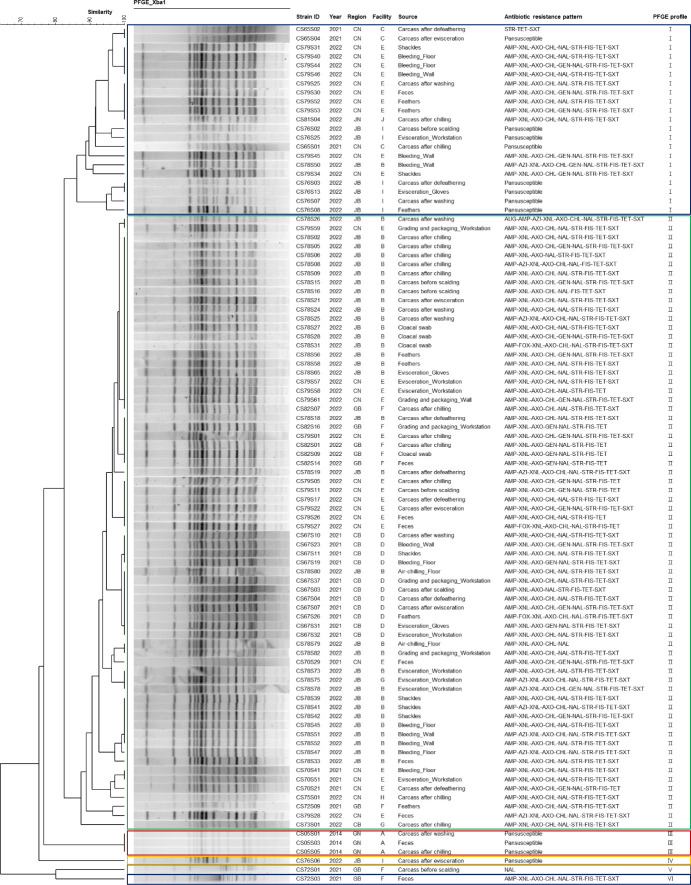
PFGE analysis and antimicrobial resistance patterns of 95 *S*. Infantis isolates from chicken slaughterhouses in South Korea (2014–2022). CB, Chungbuk; CN, Chungnam; GB, Gyeongbuk; GN, Gyeongnam; JB, Jeonbuk; JN, Jeonnam; AMP, ampicillin; AUG, amoxicillin-clavulanic acid; AXO, ceftriaxone; AZI, azithromycin; CHL, chloramphenicol; CIP, ciprofloxacin; FIS, sulfisoxazole; FOX, cefoxitin; GEN, gentamicin; NAL, nalidixic acid; STR, streptomycin; SXT, trimethoprim-sulfamethoxazole; TET, tetracycline; XNL, ceftiofur.

Six PFGE profiles were identified with ≥85% similarity, with most isolates from 2021 to 2022 demonstrating identical or closely related PFGE profiles, distinct from those in 2014 (Fig. 2). Based on the antimicrobial resistance patterns and PFGE profiles, we hypothesized the pESI plasmid acquisition of the isolates in 2021 and 2022. To test this, we conducted plasmid profiling of 38 selected isolates from each PFGE profile (3, 8, and 27 isolates from 2014, 2021, and 2022, respectively). This analysis indicated that one nalidixic acid-resistant isolate (CS72S01) and all MDR isolates, except for one isolate (CS79S34) from 2021 to 2022, harbored a plasmid >200 kb, similar in size to the pESI plasmid that was absent in all of the isolates from 2014 (Table S4). Using specific primers from pESI-encoded genes, we established that one nalidixic acid-resistant isolate (CS72S01) and all MDR isolates harbored a putative pESI plasmid (Table S4). We performed conjugation experiments on 30 strains suspected of harboring the pESI plasmid, which were identified through plasmid profiling and PCR screening of pESI genes. Among these donor strains, only the pESI plasmids from 27 strains (90.0%) were successfully transferred to recipient *E. coli* RG488 (Table S4).

### *S*. Infantis isolates of ST32 and their distinct cgMLST clusters based on the presence of the pESI plasmid

**Fig 3:**
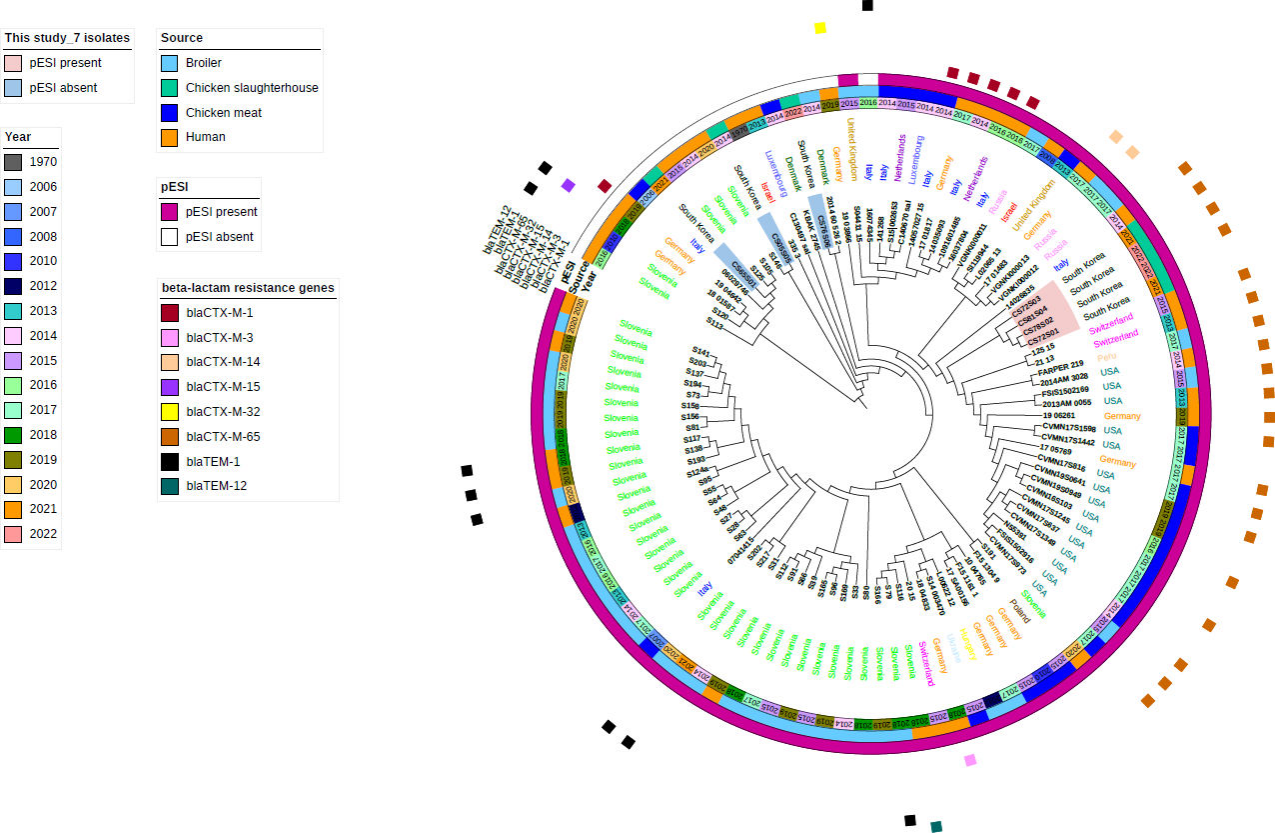
cgMLST tree of 7 *Salmonella* Infantis strains isolated from chicken slaughterhouses in this study and 94 *S*. Infantis isolates obtained from broiler, chicken meat, and humans in 15 countries registered in public databases (NCBI or ENA; data collected on 20 December 2023). Circular representation of the phylogeny was visualized using iTOL (http://itol.embl.de/) ignoring branch length. The tree includes information on the isolates, such as isolation year, source, and presence of pESI and beta-lactam resistance genes.

All seven isolates submitted to WGS belonged to the ST32 of MLST. The cgMLST tree of the seven isolates from this study and 94 foreign isolates collected from public databases demonstrated that the pESI^+^ isolates clustered with the exclusion of pESI^−^ isolates (Fig. 3). The pESI^−^ isolates (CS65S01, CS05S05, and CS76S06) formed a cluster with the pESI^−^ strains isolated from Slovenia, Germany, Italy, Luxembourg, and Denmark, including the representative pre-emergent *S*. Infantis strain 335-3. The majority of these isolates lacked the ESBL genes. In contrast, the pESI^+^ isolates (CS72S03, CS81S04, CS78S02, and CS72S01) were clustered with pESI^+^ strains isolated from various countries, including the representative ESI SI119944, which primarily harbored the *bla_CTX-M-65_* gene (Fig. 3; Table S1). There was no evident genetic association based on the source or year of strain isolation.

### pESI plasmid determining extended-spectrum cephalosporin resistance and multidrug resistance

The genetic characteristics based on WGS, including ARG profiles, plasmid incompatibility group, and pMLST types are presented in Table 2; Table S1. The pESI^−^ strains CS05S05 isolated in 2014, CS65S01 isolated in 2021, and CS76S06 isolated in 2022 were phenotypically pan-susceptible to the tested antibiotics. However, genes related to aminoglycoside resistance (*aac(6′)-ly*, *acrD*, and *kdpE*), fluoroquinolone resistance (*emrA*, *emrB*, *emrR*, and *mdtK*), and nitroimidazole resistance (*msbA*) were identified in the chromosomal loci of these isolates using WGS analysis. The pESI^+^ isolates, including CS72S03 isolated in 2021 and CS78S02 and CS81S04 isolated in 2022, were MDR and resistant to six classes of antimicrobials: beta-lactam, phenicols, quinolones, aminocyclitols, folate pathway inhibitors, and tetracyclines, whereas CS72S01, isolated in 2021, was resistant to nalidixic acid. WGS analysis of the MDR-pESI^+^ isolates revealed that the Incl1 pMLST on the pESI plasmid harbored four genes (*ardA_11*, *pilL_3*, *songS_14*, and *trbA_8*), whereas CS72S01 harbored three genes (*ardA_11*, *songS_14*, and *trbA_8*). Regarding pESI-encoded ARGs, CS72S01 harbored only *APH(3′)-Ia* and *dfrA14* (aminoglycoside resistance), whereas the MDR-pESI^+^ isolates (CS72S03, CS78S02, and CS81S04) harbored *floR* (phenicol resistance), *tetA* (tetracycline resistance), *AAC(3)-IV*, *aadA1*, *ANT(3′′)-IIa*, and *APH(4)-Ia* (aminoglycoside resistance), and *sul1* and *dfrA* (folate pathway inhibitor resistance). All these isolates were phenotypically resistant to cephalosporins, commonly harboring the ESBL gene *bla_CTX-M-65_* (Table 2; Table S1). Additionally, all the pESI plasmids harbored virulence- and fitness-associated traits, including the iron acquisition system (*ybt* and *irp*) and two chaperone-usher fimbrial gene clusters (*fae*) (Table S1).

**TABLE 2:** Phenotypic and genotypic characteristics of seven representative *S*. Infantis isolates from each PFGE profile

Strain ID	Year	Facility	Source	PFGE profile	Antimicrobial resistance pattern (phenotype)	WGS analysis				
						Antimicrobial resistance genes (genotype)		MLST ST	pMLST (Incl1)	Plasmid incompatibility group
						Chromosome	Plasmid			
CS05S05	2014	A	Carcass after chilling	III	Pansusceptible	*aac(6′)-ly, acrD, kdpE, emrA, emrB, emrR, mdtK, msbA*	NF^*a*^	32	NF	NF
CS65S01	2021	C	Carcass after chilling	I	Pansusceptible	*aac(6′)-ly, acrD, kdpE, emrA, emrB, emrR, mdtK, msbA*	NF	32	NF	NF
CS72S01	2021	F	Carcass before scalding	V	NAL	*aac(6′)-ly, acrD, kdpE, emrA, emrB, emrR, mdtK, msbA*	*APH(3’)-Ia, dfrA14*	32	*ardA_11, trbA_8, sogS_14*	IncFIB(pESI)
CS72S03	2021	F	Feces from crates	VI	AMP-XNL-AXO-CHL-NAL-STR-FIS-TET-SXT	*aac(6′)-ly, acrD, kdpE, emrA, emrB, emrR, mdtK, msbA*	*CTX-M-65, floR, tet(A), AAC (3)-IV, aadA1, APH(3’)-Ia, ANT(3’’)-IIa, APH (4)-Ia, sul1, dfrA14*	32	*ardA_11, trbA_8, sogS_14, pilL_3*	IncFIB(pESI)
CS76S06	2022	I	Carcass after evisceration	IV	Pansusceptible	*aac(6′)-ly, acrD, kdpE, emrA, emrB, emrR, mdtK, msbA*	NF	32	NF	NF
CS78S02	2022	B	Carcass after chilling	II	AMP-XNL-AXO-CHL-NAL-STR-FIS-TET-SXT	*aac(6′)-ly, acrD, kdpE, emrA, emrB, emrR, mdtK, msbA*	*CTX-M-65, floR, tet(A), AAC (3)-IV, aadA1, ANT(3’’)-IIa, APH (4)-Ia, sul1, dfrA14*	32	*ardA_11, trbA_8, sogS_14, pilL_3*	IncFIB(pESI)
CS81S04	2022	J	Feces from crates	I	AMP-XNL-AXO-CHL-NAL-STR-FIS-TET-SXT	*aac(6′)-ly, acrD, kdpE, emrA, emrB, emrR, mdtK, msbA*	*CTX-M-65, floR, tet(A), AAC (3)-IV, aadA1, APH(3’)-Ia, ANT(3’’)-IIa, APH (4)-Ia, sul1, dfrA14*	32	*ardA_11, trbA_8, sogS_14, pilL_3*	IncFIB(pESI)

^*a*^ NF, not found.

### Comparative analysis of pESI plasmids

**Fig 4:**
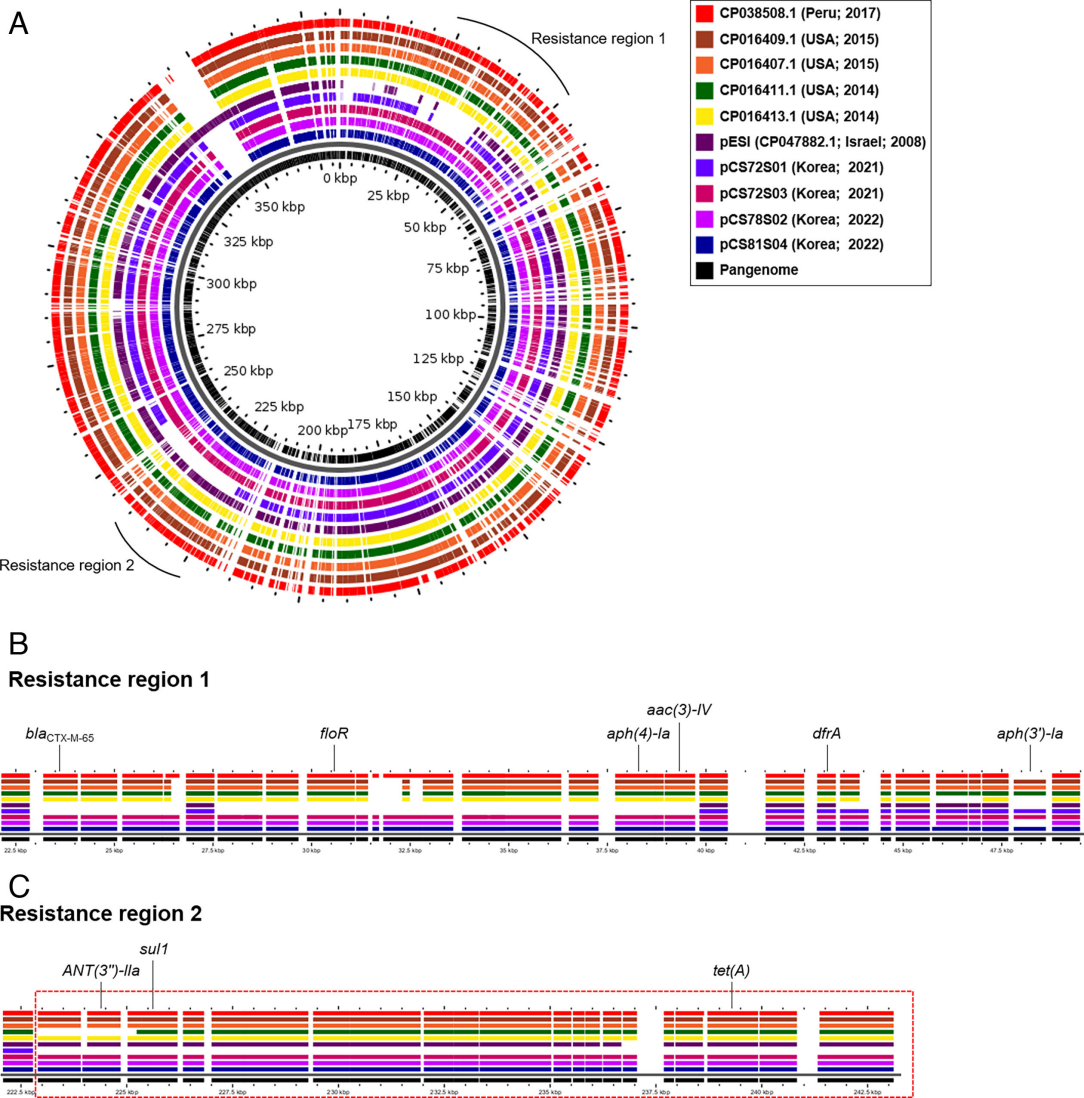
Pangenome analysis of four South Korean pESI plasmids (CS72S01, CS72S03, CS78S02, and CS81S04) compared with the original Israeli pESI and other pESI-like plasmids. (**A**) Pangenome analysis; (**B**) resistance region 1; (**C**) resistance region 2.

The complete pESI plasmid sequences of the Korean pESI strains (CS72S01, CS72S03, CS78S02, and CS81S04) revealed that most of the acquired ARGs were plasmid-borne and comprised two resistance regions (Fig. 4). Among the four South Korean strains, CS72S01, which was resistant to nalidixic acid, had *dfrA* and *aph(3′)-*I*a* located in resistance region 1 and lacked ARGs in resistance region 2. The MDR and cephalosporins-resistant strains (CS72S03, CS78S02, and CS81S04) harbored the ESBL gene *bla_CTX-M-65_* in resistance region 1, along with *floR*, *aph (4)-Ia*, *aac (3)-IV*, and *dfrA*. Additionally, the resistance region 2 of these plasmids harbored *ANT(3′′)-IIa (aadA1*), *sul1*, and *tet(A*).

The comparative analysis of the pESI sequences revealed that all South Korean pESI plasmids are genetically related (Fig. 4). This was confirmed by the high average nucleotide identity based on the ANIm (99.99% identity over coverage of approximately 84.25%–100%) (Table S5). Specifically, the pESI plasmids of the MDR and cephalosporin-resistant isolates (CS72S03, CS78S02, and CS81S04) were most similar to the USA pESI plasmids (pFSIS1502916, pFSIS1502169, pN55391, and pVCM44454) that harbored the *bla_CTX-M-65_* gene, rather than the original Israeli pESI plasmid (Fig. 4), with a minimum identity of 99.98% (Table S5).

## DISCUSSION

*S*. Infantis is currently the most prevalent serovar isolated from fresh poultry meat and broiler flocks in various countries (3, 7–9, 11, 49). Human *S*. Infantis infections are often associated with poultry meat (3, 49).

In this study, the annual *Salmonella*-positive rates ranged from 17.5% to 59.9% (average 37.4%), comparable to those in chicken slaughterhouse samples from Brazil (3.6%), USA (11.5%), Taiwan (25.1%), South Korea (28%), and East China (57%) (50–54). In this study, *S*. Infantis was identified in 9.3% of *Salmonella*-positive samples in 2014, remained undetected from 2015 to 2020, and re-emerged as the most frequently identified serovar in 2021 and 2022, with rates of 54.7% and 75.5%, respectively. This emergence aligns with global observations, where *S*. Infantis is increasingly recognized as a dominant serovar. Human infections associated with this serovar increased by 167% in the United States between 2001 and 2016 (55). Additionally, in the European Union, Infantis has become the most prevalent serovar in broiler flocks and broiler meat, accounting for 56.7% of all *Salmonella* isolates in broiler meat in 2018 (56, 57). Similarly, in Japan, 72.2% of *Salmonella* isolates from ground chickens were identified as Infantis, and in Ecuador, the prevalence in broilers reached 84% (58, 59).

*S*. Infantis is increasingly viewed as a public health threat owing to its widespread distribution across various countries and high levels of antimicrobial resistance (6, 7, 12, 44, 60). In this study, the *S*. Infantis strains isolated in 2014 were susceptible to all tested antibiotics. However, the majority of strains isolated in 2021–2022 were MDR, resistant to critically important antimicrobials, and third-generation cephalosporins. This indicated a significant shift in the antibiotic resistance patterns of *S*. Infantis. Additionally, the U.S. National Antimicrobial Resistance Monitoring System reported that MDR *S*. Infantis was observed in <1% of retail chicken samples in the United States in 2014 but increased to approximately 30% by 2019 (44). Moreover, in Europe, approximately 70% of the *S*. Infantis isolates from broiler meat were MDR in 2016 (60). These figures highlight a significant increase in antimicrobial resistance among *S*. Infantis strains, indicating an increasing public health challenge associated with poultry consumption.

The spread of the pESI plasmid has been a significant factor in the increase of MDR *S*. Infantis (6, 7, 12). We confirmed that the antimicrobial-resistant isolates from 2021 to 2022 harbored the pESI plasmid through plasmid profiling and PCR screening, using specific primers from pESI-encoded genes. Additionally, we observed that the majority (90.0%) of these isolates can transfer the pESI plasmid to other bacterial strains through conjugation. These findings indicate that, as observed in other countries, South Korea has experienced the emergence of *S*. Infantis strains carrying the conjugative pESI plasmid, identified in diverse samples from chicken slaughterhouses, including cloacal swabs from livestock, environmental samples, and chicken carcasses.

We hypothesize that the emergence and increased isolation rates of the MDR and third-generation cephalosporin-resistant *S*. Infantis in South Korean chicken slaughterhouses since 2021 result from several factors. First, the import of the parent stock contaminated with *S*. Infantis harboring the pESI plasmid likely introduced this pathogen into the country. Additionally, since 2002, ceftiofur has been administered to 1-day-old chicks in South Korea in conjunction with vaccines for Marek’s disease and infectious bursal disease, either through *in ovo* or subcutaneous injection (61). The use of ceftiofur on farms has selectively facilitated the survival and spread of these resistant strains throughout the chicken production chain. Although this study did not analyze *S*. Infantis isolates from humans in South Korea, it indicates that the presence of MDR *S*. Infantis harboring pESI at chicken production stages can potentially pose a public health threat throughout the food chain.

Since the first report of an Israeli *S*. Infantis isolate 119944 harboring the pESI megaplasmid in 2008 (21), various countries have conducted detailed analyses of *S*. Infantis isolates with pESI plasmids using the WGS tools (11, 14, 15, 17, 22, 26, 44, 45). Additionally, we performed WGS analysis on seven representative *S*. Infantis isolates (three pESI^−^ and four pESI^+^ isolates) to reveal their genetic molecular characteristics and genetic relatedness when compared to *S*. Infantis strains from other countries and sources, including broilers, chicken meat, and humans. In this study, all isolates were identified as MLST-type ST32, regardless of pESI plasmid possession. ST32 is a highly conserved sequence type of *S*. Infantis (62) and is the predominant MLST type isolated from various sources (broilers, pigs, cattle, food, and humans) across different countries (8, 12, 14, 22, 63–65). Additionally, *S*. Infantis isolates from broilers and humans in Slovenia were predominantly identified as ST32, regardless of pESI plasmid possession (26). In this study, although all isolates belonged to a single sequence type, the *S*. Infantis population exhibited heterogeneity in molecular relatedness at the cgMLST level, which was affected by the presence of the pESI plasmid. This finding aligns with similar research conducted in Slovenia (26). In this study, pESI^−^ isolates formed a genetic cluster with pESI^−^ isolates from various countries, including the pre-emergent *S*. Infantis strain 335-3. In contrast, pESI^+^ isolates formed a similar clonal cluster with minor genetic differences, including isolates from the USA, Switzerland, Peru, and Germany, specifically those harboring the *bla_CTX-M-65_* gene from the ESI group. Notably, these isolates, derived from broilers, chicken meat, and human sources, indicate that the pESI^+^ isolates in this study can pose a potential public health threat throughout the food chain. To further substantiate this, additional collection and genetic studies of *S*. Infantis derived from humans, chicken meat, broilers, and farm environments in South Korea are necessary.

The pESI plasmids frequently harbor resistance genes against at least three classes of antimicrobials (sulfonamides, tetracyclines, and trimethoprim), with variable possession of aminoglycoside-resistance genes, such as *aadA1*, and survival- and virulence-associated genes (9, 11, 21, 22). In the present study, although several virulence genes were identified in all seven isolates analyzed through WGS, the four pESI^+^ isolates specifically harbored the *Yersinia* high-pathogenicity island toxin (Ybt) system and fimbriae-coding genes. These genes are involved in enhancing *Salmonella* survival under adverse conditions and increasing its adhesion to human and animal cells. The Ybt operon, involved in iron acquisition, increases the ability of *Salmonella* to survive under low-iron conditions (66). Additionally, the pESI^+^ isolates contained few or all the typical pESI-associated genes, including *aadA1*, *dfrA14*, *sul1*, and *tet(A*). Moreover, their pESI plasmids harbored ARGs of 2–6 different antibiotic classes. Notably, among the four pESI^+^ isolates, the pESI^+^ isolate (CS72S01) that exhibited phenotypic resistance only to nalidixic acid had fluoroquinolone resistance genes in the chromosome but lacked plasmid-related phenotypic resistance genes in the plasmid. In contrast, the three isolates (CS72S03, CS78S02, and CS81S04) with an MDR phenotype had antibiotic resistance genes related to their phenotypic antibiotic resistance in the plasmid. This indicates that plasmid-derived resistance genes are crucial for determining the antibiotic resistance phenotypes of the isolates.

The phenotypic and genotypic resistance profiles were consistent with the previously described pESI-harboring *S*. Infantis isolates from other countries (11, 12, 22). ESBL-producing bacteria are resistant to ampicillin and extended-spectrum third-generation cephalosporins (67). Three MDR pESI^+^ *S*. Infantis isolates (CS72S03, CS78S02, and CS81S04) from this study contained the *bla_CTX-M-65_* gene, which encodes CTX-M β-lactamase. The pESI^+^ isolates in this study were genetically related to pESI^+^ isolates from Italy, the USA, Germany, and Slovenia that also harbored the *bla_CTX-M-65_* gene, as indicated by cgMLST analysis. Third-generation cephalosporins are among the few treatment options available for severe cases of human salmonellosis. Therefore, treatment can be further complicated by the integration of additional resistance genes into pESI. The high genome plasticity of pESI plasmids may facilitate the evolutionary adaptation of *S*. Infantis to various environments and selection pressures (21, 24). Consequently, the emergence of *S*. Infantis harboring the *bla_CTX-M-65_* integrated megaplasmid in abattoirs is a public health concern because it can potentially be transmitted to humans through the food chain. Notably, the pESI plasmid of our MDR and ESBL-producing *S*. Infantis isolates had *bla_CTX-M-65_-floR-aph (4)-Ia-aac (3)-IV* inserted in the resistance region 1 of the original Israeli pESI plasmid. This indicated that the Korean pESI has integrated various ARGs, including *bla_CTX-M-65_*, into the original pESI backbone. Additionally, when compared to representative *bla_CTX-M-65_*-positive pESIs from Peru and the USA, the pESI from South Korea in this study was most similar to the pESI of *S*. Infantis isolated from chicken carcass, chicken ceca, retailed chicken, and human clinical samples in the USA. The ARGs in resistance regions 1 and 2 were identical, and despite variations in the tested antibiotics, they exhibited phenotypic resistance to the same antibiotic classes (17). The MDR *S*. Infantis clones prevalent in the chicken production chain in South Korea are highly similar to the pESI-harboring *S*. Infantis clones that are widespread globally. This indicates that monitoring bacterial antibiotic resistance should not be limited to within the country but should also consider global patterns. Additionally, broiler farms and chicken slaughterhouses should implement research and management programs for antibiotic alternatives, such as *S*. Infantis-targeting bacteriophages or probiotics, to reduce the spread of MDR *S*. Infantis.

### Conclusion

In this study, we observed a significant increase in *S*. Infantis and MDR *S*. Infantis in chicken slaughterhouses in South Korea since 2021 through the national *Salmonella* monitoring program. Using WGS, we observed that the rise in MDR and cephalosporin-resistant *S*. Infantis is largely driven by the acquisition of the pESI megaplasmid harboring the ESBL determinant *bla_CTX-M-65_*. Genetic analysis demonstrated that our pESI^+^ isolates closely resemble those identified in broilers, chicken meat, and a human case from various countries, indicating a global public health threat posed by this strain. Our findings highlight not only the urgent need to control *S*. Infantis at the primary production level, including broiler farms and chicken slaughterhouses, but also for international cooperation and continuous genomic surveillance of these strains as part of a comprehensive One Health strategy. Our study contributes to the growing body of evidence that emphasizes the transboundary nature of antimicrobial resistance, stressing the importance of global coordination in mitigating the spread of MDR pathogens.

## Data Availability

All the WGS read data (Table S1) and pESI plasmid sequences (Table S2) obtained in this study have been deposited in the NCBI Sequence Read Archive (SRA) under BioProject accession number PRJNA1145000.

## SUPPLEMENTAL MATERIAL

Supplemental material spectrum.02955-24-s0001.xlsx Tables S1 to S5.

ASM does not own the copyrights to Supplemental Material that may be linked to, or accessed through, an article. The authors have granted ASM a non-exclusive, world-wide license to publish the Supplemental Material files. Please contact the corresponding author directly for reuse.
